# Nutritional Adaptations in Elite Soccer Referees: First Evidence and Perspectives

**DOI:** 10.1515/hukin-2015-0036

**Published:** 2015-07-10

**Authors:** Lore Metz, Thomas Deleuze, Bruno Pereira, David Thivel

**Affiliations:** 1 Clermont University, Blaise Pascal University, Laboratory of the Metabolic Adaptations to Exercise under Physiological and Pathological Conditions (AME2P), Clermont-Ferrand, France.; 2 CRNH-Auvergne, Clermont-Ferrand, France.; 3 PERF ARBITRAGE, Blaise Pascal University, Aubière cedex, France.; 4 Clermont-Ferrand University Hospital, Biostatistics unit (DRCI), Clermont-Ferrand, France.

**Keywords:** soccer referees, energy intake, stress, appetite

## Abstract

Although the physiological cost of refereeing has been already studied in the literature, especially in soccer umpires, it remains unknown whether referees spontaneously adapt their energy intake during game days. Six national soccer referees completed 24-hour dietary recalls (assisted by the SU.VI.MAX copybook) during a control day (CON) and a day with a game (GAME). The stress level and hunger feelings were assessed using visual analogue scales. Total energy intake, energy derived from macronutrients and energy intake at each meal were analyzed using the Bilnuts nutrition software. Total daily energy intake was not significantly different between conditions (CON: 2270 ± 535 vs. GAME: 2782 ± 293). Energy derived from fat and protein was not different between conditions but the participants ingested more calories derived from carbohydrates during the GAME day (45.5 ± 5.9% vs. 54.9 ± 5.5%, respectively, p<0.05). The calories ingested during snacking were significantly increased during GAME compared with CON (p<0.05). The stress level was significantly higher during GAME and especially before the breakfast, lunch and snack (p<0.05). Hunger feeling was not different between conditions. Referring leads to nutritional adaptations in elite soccer umpires, who tend to increase their energy intake mainly during snacking, by increasing their carbohydrate consumption.

## Introduction

As for physical activity, nutrition is a key point in health promotion and a fundamental parameter for sports performance. Nutritional strategies are today a part of physical conditioning and training in elite athletes. Referees are nowadays considered as real athletes since they follow regular and intensive training programs to reach a high level of performance. In soccer, referring is closely related to endurance exercises with low-to-high intensity phases of running. It has been recently shown that soccer umpires can run up to 10 km (usually between 5 and 10 km, with main field referees usually close to 10 km) during a game, altering low to high rates of speed (Catterall et al., 1993; Johnston and McNaughton, 1994; Krustrup and Bangsbo, 2001; Mallo et al., 2007), which is pretty similar to what is observed in midfield players (Bangsbo et al., 1991; Mohr et al., 2003; Rienzi et al., 2000; Tumilty, 1993). Some studies have concentrated on the energy cost of a soccer game in referees and collectively show that main field soccer umpires expend approximately 800 kilocalories during a single game (Da Silva et al., 2008). Although it has been shown that soccer referees have significant aerobic energy expenditure throughout a game, an important number of episodes of anaerobic energy turnover have been identified indicating that soccer referring is a highly intermittent exercise modality (Krustrup and Bangsbo, 2001). Altogether, the muscular and cognitive (attentional and decisional tasks) activity of referees leads to an important glycogen depletion that could alter their performances during a game. Nutritional guidelines have been proposed in endurance athletes with the American College of Sports Medicine recommending for instance intakes of about 6 to 10 g·kg-1 of body weight for carbohydrates (CHO), 1.2 to 1.4 g·kg-1 for the protein intake and the equivalent of 20 to 35% of the total energy intake for fat (Rodriguez et al., 2009), but data are missing regarding referees’ nutritional adaptations and needs.

To date, we found only one study directed at referees’ eating behaviors (Teixeira et al., 2014). In their work, Teixera and collaborators compared the energy intake between main and assistant soccer referees (Teixeira et al., 2014). Although they did not register any differences between the two groups, they observed in both main and assistant umpires that the carbohydrate intake was above the recommended values and found a deficiency of their micronutrients intake (Teixeira et al., 2014). Although Teixeira et al. (2014) explored the referees’ energy intake before, during and after a game, they did not compare those nutritional behaviors with a control period (without game).

The aim of the present work was to determine whether the energy intake (chronobiology, quantitative and qualitative energy intake) and appetite feelings are modified in response to a game in elite soccer referees.

## Material and Methods

After a first visit when the referees were instructed how to fill in the questionnaires, they participated in two experimental sessions completed in a randomized order: i) a control session corresponding to a day with no game (CON); ii) a day with a game to umpire (GAME). Those two experimental sessions were separated by at least 7 days to avoid any interference in the measurements. The participants were asked not to engage in any physical activity during the control day. During the whole day, their total energy intake, food preferences, meal chronobiology, hunger and stress feelings were assessed.

### Subjects

For the pilot study, six national soccer referees aged 22.6 ± 6.8 years participated in the research.

### Procedures

This project was conducted in accordance with the local ethical policies and each subject signed an informed consent form to participate in the study.

### Measures

***Energy intake.***The participants were asked to complete 24 hours dietary recall during both CON and GAME days. They were asked to indicate all the consumed calories and the ingestion time. In order to facilitate the completion of the recall and to improve its accuracy, the SU.VI.MAX copybook was provided to each participant. Based on pictures that represent a large variety of food depending on their volume, the SU.VI.MAX allows a better estimation of the caloric intake. This method had been developed and previously used as accurate in different populations (Hercberg et al., 2004). The energy intake and food choices were qualitatively and quantitatively assessed using the Bilnuts software (Bilnut 4.0 SCDA Nutrisoft software, France).

***Hunger and stress feelings.*** Hunger feeling was assessed before each meal using a Visual Analogue Scale on which the participants had to indicate their sensation from 0 to 10. The 0 score represents the “absence of hunger sensation” and 10 stands for “very hungry”. Similarly, the subjects’ stress level was also evaluated using visual analogue scales from 0 to 10; 0 representing an “absence of stress” and 10 “very stressed”. The VAS method had been validated in several populations (Flint et al., 2000).

### Statistical analyses

Data are expressed as mean ± standard deviations. Since the sample size was limited the data were not normally distributed (after verification by means of a Smirnov-Kolmogorof normality test) and non-parametric tests were used. The total daily energy intake, energy-derived from Fat, Protein and CHO were compared between CON and GAME days using the Wilcoxon signed rank test for a paired t-test. The energy intake, at each meal was compared using the Friedman non-parametric procedure (condition × meal). Area Under the Curves (AUC) were calculated for stress and hunger scores and the Friedman test was performed (meal × condition). The level of significance was set at p<0.05. The z statistical value has also been obtained and effect sizes calculated using Hedge’s calculation that had been developed for small sample size using paired analyses with effect size = 0.2 as a low effect, 0.5 as a medium effect, 0.8 as a high effect and 1.3 as a very large effect.

## Results

**Figure 1:**
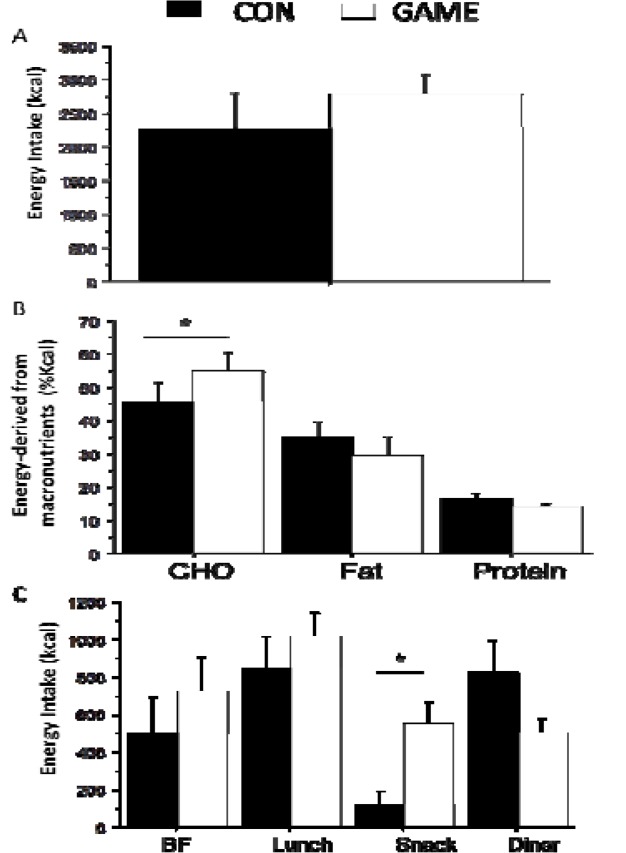
(A) Total daily energy intake during GAME and CON days; (B) Total daily energy derived from macronutrients between conditions; (C) Energy intake during each meal on GAME and CON days. CON: Control day; GAME: Game day; CHO: Carbohydrate; BF: Breakfast; *p<0.05.

Although the total daily energy intake was higher during the GAME day, the difference was not statistically different when compared with CON as shown in Figure 1A (CON: 2270 ± 535 vs. GAME: 2782 ± 293 kcal, respectively). The energy derived from fat and protein was not significantly different between conditions but the energy ingested derived from CHO was significantly increased on the GAME day when compared with CON (*p*=0.01; *z* = −2.0; *effect size* = 0.7; Figure 1B). Although Figure 1C illustrates a slightly higher energy intake during breakfast (BF) (501 ± 191 vs. 727 ± 180 kcal, respectively), lunch (842 ± 176 vs. 1020 ± 122 kcal, respectively) and snack time (120 ± 75 vs. 547 ± 123 kcal, respectively) during the GAME day when compared with CON, this is only significant for snacking (*p*=0.01; *z* = −1.9; *effect size* = 1.3). The energy intake at lunch time was lower during the GAME day when compared with CON but this did not reach the level of significance (823 ± 171 vs. 499 ±73, respectively).

**Figure 2:**
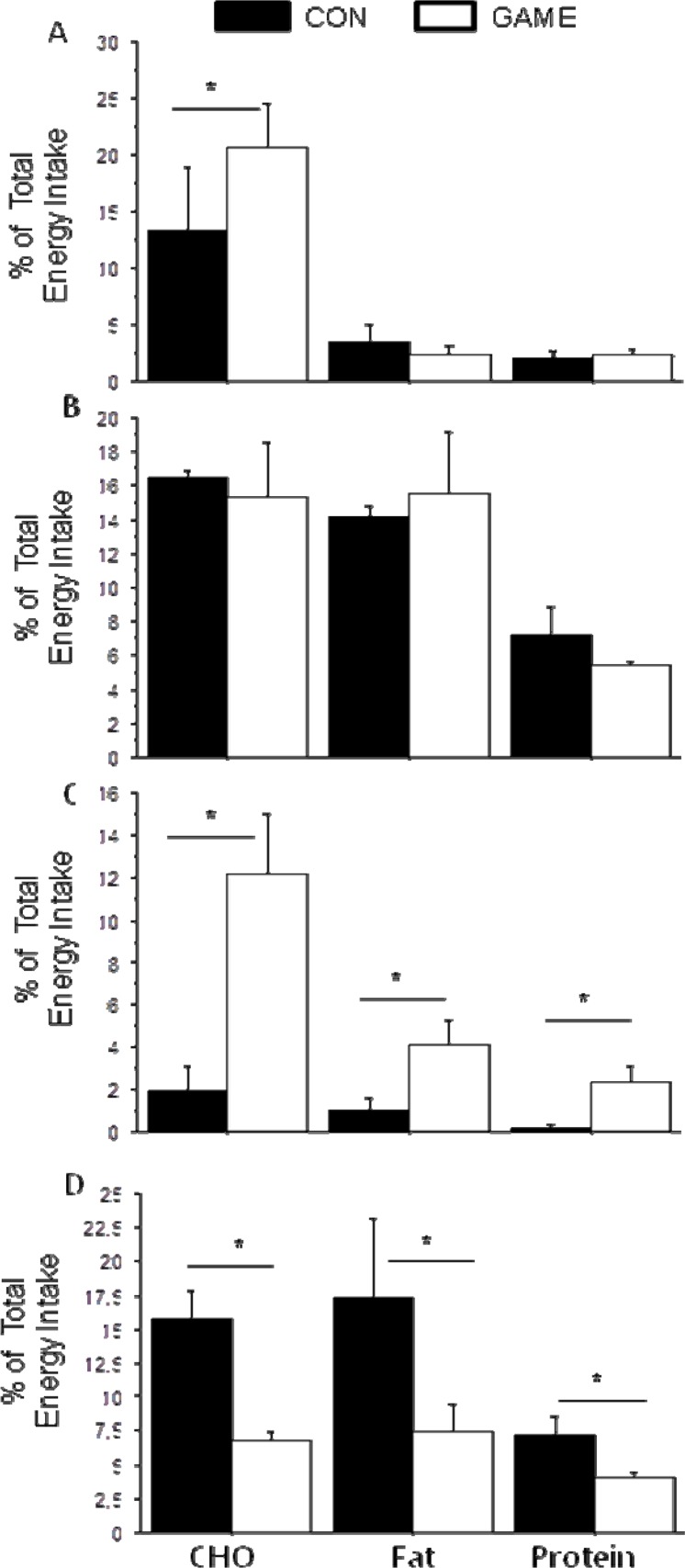
Carbohydrate, Fat and Protein intake during (A) Breakfast; (B) Lunch; (C) Snack; (D) Dinner on GAME and CON days. Values are expressed as percentage of the Total Energy Intake. CON: Control day; GAME: Game Day; CHO: Carbohydrate; BF: Breakfast; *p<0.05

The carbohydrate (CHO), fat and protein intake during each meal is shown in Figure 2. There is an increase in the CHO intake during breakfast (*p*=0.04; *z* = −2.1; *effect size* = 0.6), snack (*p*=0.02; *z* = −1.4; *effect size* = 1.6) and a decrease during dinner (*p*=0.008; *z* = 2.2; *effect size* = 2.6) on the GAME day. The fat and protein intake increased during snack *(p*=0.05; *z* = −2.1; *effect size* = 1.7 and *p*=0.03; *z* = −1.4; *effect size* = 2.0) and decreased significantly at diner (*p*=0.05; *z* = −1.3; *effect size* = 0.8 and *p*=0.05; *z* = 0.7; *effect size* = 1.1) during the GAME day.

**Figure 3:**
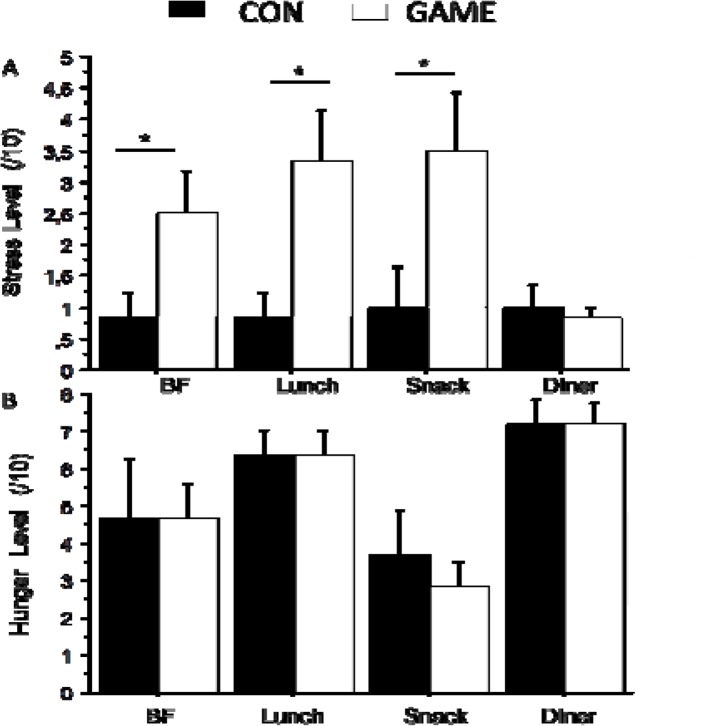
(A) The level of stress before each meal during experimental conditions; (B) Hunger feeling before each meal during CON and GAME days. CON: Control day; GAME: Game day; BF: Breakfast; *p<0.05.

As shown in Figure 3A, the level of stress experienced before each meal was significantly different between GAME and CON and especially significantly higher before BF (*p*=0.03; *z* = −1.9; *effect size* = 1.2), lunch (*p*=0.01; *z* = −2.1; *effect size* = 1.6) and snack (*p*=0.01; *z* = −2.2; *effect size* = 1.2) on the GAME when compared with the CON day. The hunger score was not different between both conditions (Figure 3B)

## Discussion

This pilot study is the first to investigate the nutritional adaptations (chronobiology, quantitative and qualitative energy intake) during a game day in elite soccer referees. Although, they do not reach the level of significance (which is mainly due to the reduced sample size), our results show an increased total energy intake during a game day compared with a control day. Interestingly, this higher energy intake has two main explanations.

First, referees significantly increased their energy intake derived from carbohydrate during the game day without modifying their fat and protein ingestion. Although these results are based on a relatively small sample size, its medium to large effect size confirms its accuracy (effect size = 0.7). While the carbohydrate intake during the control day (mean 45%) respects the actual recommendations and matches with some recent epidemiological results in the general French populations (44%, (Aliments, 2009)), it reaches about 54% (+10%) during the game day, and is then in accordance with the ACSM guidelines for athletes (Rodriguez et al., 2009). This increased carbohydrate consumption joins up with the results recently obtained by Teixera and collaborators in both main and assistant soccer referees (Teixeira et al., 2014). Importantly, it underlines that elite soccer referees tend to adapt their energy intake to their practice. This result effectively suggests their ability to adapt the relative contribution of each macronutrient (reduction, albeit non-significant of the proportion of energy derived from the fat and protein intake and an increase of the CHO intake percentage) in response to a physiological and energetic demand (umpiring a game in the present case). Such an increase in carbohydrate might effectively reflect the need to compensate for the important glycogen depletion induced by the game by refilling stores, insuring a better recovery process.

The second important finding concerns the chronobiology of their energy intake. Our results effectively show that during a game day, elite soccer umpires modify the repartition of their daily energy intake by decreasing (even though it does not reach the level of significance) the breakfast and lunch time energy intake and by significantly (the very large effect size (1.3) supports the validity of this results) increasing their snacks consumption (in the present study the snacks were taken after the game). Snacks effectively represent 3% of the total daily energy intake during the control day and reach up top 19% during the game day. Interestingly, the increased caloric intake observed during snacking seems to correspond and then compensate for the reduction observed at dinner time (from 40 to 20% of the daily energy intake). This might suggest a need for a rapid repletion of the game energy demand.

As illustrated in Figure 2B, the nutritional adaptations observed during the game day are not accompanied by any modification of hunger. Although it might be surprising that hunger and effective energy intake modifications are not matched, it is in accordance with the actual literature reporting an uncoupled response of appetite feelings and the caloric intake to exercise in both lean and obese, children and adult individuals (Hubert et al., 1998; Thivel and Chaput, 2014). The stress level, however, has been shown to affect the energy intake and appetite control (for review see McEwen, 2008). Our results show that the stress level (assessed by visual analogue scales) is significantly increased before breakfast, lunch and snack time (with large effect size of 1.2, 1.6 and 1.2, respectively, highlighting the validity of the results). Although the reduced sample size does not allow us to obtain any significant correlation between the stress level and effective energy intake, it might have led to the higher energy intake observed during the game day. Importantly it would be useful to assess the stress level by means of physiological parameters (such as salivary cortisol) and to apply VAS as indicators of attentional demand and concentration. Effectively, cognitive and attentional work could be an explaining factor for the observed increased energy intake and especially CHO consumption, as previously found in adults (Chaput et al., 2008; Chaput and Tremblay, 2007), particularly through the glucostatic theory involved in energy intake control (Chaput and Tremblay, 2009).

Although this work is a pilot study with a reduced sample size, it suggests for the first time that elite soccer umpires modify the chronobiology and nature of their energy intake during a game day, mainly to adapt their intake to the energy demand induced by the game. The medium to very large effect sizes obtained in the present study indicate accuracy of the study results despite a reduced number of participants. This work underlines the necessity to develop further investigations using stronger sample size and methodologies, simultaneously exploring energy expenditure and intake.
